# Supplementary material for: Nano-SAR Modeling for Predicting the Cytotoxicity of Metal Oxide Nanoparticles to PaCa2
Source: Molecules. 2021 Apr 10;26(8):2188. doi: 10.3390/molecules26082188 (PMC8069170; doi:10.3390/molecules26082188)
Supplement: Supplementary file 1 [file molecules-26-02188-s001.pdf]

Electronic Supplementary Information

Nano-SAR modeling for predicting the cytotoxicity of metal oxide nanoparticles to PaCa2

Haihua Shi [1], *Yong Pan* [1,*], *Fan Yang* [1], *Jiakai Cao*[1] , *Xinlong Tan* [1], *Beilei Yuan* [1], *Juncheng Jiang*[1,2]

[1] Jiangsu Key Laboratory of Hazardous Chemicals Safety and Control, College of Safety Science and Engineering, Nanjing Tech University, Nanjing, 210009, China.

[2] School of Environment & Safety Engineering, Changzhou University, Changzhou 213164, China

* Corresponding author, E-mail: yongpan@njtech.edu.cn

Table S1: List of chemicals conjugated to nanoparticles and their corresponding cellular uptake. Reprinted with permission from ref.14. Copyright 2005, Springer Nature Group.

ID	Name	PaCa2 cellular uptake (log10 [nanoparticles]/cell pM)	Prediction by RF model
	**Training set**		
1	trifluoroacetic anhydride	4.17	1
3	pentafluoropropanoic anhydride	4.08	1
5	furan-2,5-dione	3.98	1
6	3-methylfuran-2,5-dione	3.58	0
7	3,4-dimethylfuran-2,5-dione	3.48	1
8	hexanoic anhydride	3.65	0
9	3-methyl-dihydro-furan-2,5-dione	3.64	0
10	5,5'-carbonylbis(2-benzofuran-1,3-dione)	3.51	0
11	5-nitro-2-benzofuran-1,3-dione	3.27	0
13	1,4,5,8-naphtalenetetracarboxilic anhydride	3.67	0
14	4,5,6,7-tetrafluoro-2-benzofuran-1,3-dione	3.83	1
16	4-hydroxy-2-benzofuran-1,3-dione	3.97	1
17	4-oxatricyclo[5.2.2.02,6]undec-8-ene-3,5-dione	3.90	1
18	6-chloro-2H-3,1-benzoxazine-2,4(1H)-dione	4.18	1
19	3H-2,1-benzoxathiol-3-one 1,1-dioxide	3.88	1
20	3,4-dichlorofuran-2,5-dione	3.84	1
21	S-(2,5-dioxotetrahydrofuran-3-yl) ethanethioate	3.59	0
22	5,6-dichloro-2-benzofuran-1,3-dione	4.12	1
23	4,10-dioxatricyclo[5.2.1.02,6]dec-8-ene-3,5-dione	3.82	1
27	6-nitro-1H,3H-benzo[de]isochromene-1,3-dione	3.93	1
28	tetrahydrofuro[3',4':3,4]cyclobuta[1,2-c]furan-1,3,4,6-tetrone	3.77	1
29	lauric anhydride	3.82	1
30	1,3-dioxo-1,3-dihydro-2-benzofuran-5-carboxylic acid	3.55	0
31	5-methyl-2-benzofuran-1,3-dione	3.98	1
32	4-nitro-2-benzofuran-1,3-dione	3.50	1
33	1H-isochromene-1,3(4H)-dione	3.78	1
34	dihydro-2H-pyran-2,6(3H)-dione	4.07	1
35	4,4'-ethane-1,2-diyldimorpholine-2,6-dione	3.93	1
36	2H-3,1-benzoxazine-2,4(1H)-dione	4.44	0
37	1-methyl-2H-3,1-benzoxazine-2,4(1H)-dione	3.36	1
38	4-methyldihydro-2H-pyran-2,6(3H)-dione	3.91	1
39	4,5,6,7-tetrahydro-2-benzofuran-1,3-dione	3.73	1
40	2,5-dioxotetrahydrofuran-3,4-diyl diacetate	3.91	1
41	4,5,6,7-tetrabromo-2-benzofuran-1,3-dione	3.80	0
42	hexahydro-2-benzofuran-1,3-dione	3.93	0
43	5,6-dihydro-1H-cyclopenta[c]furan-1,3(4H)-dione	3.69	0

44	iodoacetic anhydride	3.42	0
45	chloroacetic anhydride	3.63	0
46	1,7,8,9,10,10-hexachloro-4-oxatricyclo[5.2.1.02,6]dec-8-ene-3,5-dione	3.47	1
47	Palmitic anhydride	3.55	1
48	6-amino-1H,3H-benzo[de]isochromene-1,3-dione	3.64	1
49	Decanoic anhydride	4.03	1
51	4-oxatricyclo[5.2.1.02,6]decane-3,5-dione	3.94	1
52	1H,3H-benzo[de]isochromene-1,3-dione	3.96	1
54	4,5,6,7-tetrachloro-2-benzofuran-1,3-dione	3.83	1
55	4,7-dichloro-2-benzofuran-1,3-dione	3.90	1
56	3,3-dimethyldihydro-2H-pyran-2,6(3H)-dione	3.94	0
57	pentan-1-amine	3.78	1
58	4-methylpentan-2-amine	3.85	1
59	3-amino-6-(hydroxymethyl)cyclohexane-1,2,4-triol	3.36	1
60	hexan-1-amine	3.75	1
61	2-methylpropan-2-amine	3.86	1
62	2-methylpropan-1-amine	3.72	0
63	2,2-dimethylpropan-1-amine	3.75	1
66	2-methylbutan-2-amine	4.07	0
67	ethane-1,2-diamine	3.46	0
68	pentadecan-1-amine	4.06	1
70	butane-1,4-diamine	3.48	1
71	hexane-1,6-diamine	3.62	0
72	2-ethylhexan-1-amine	3.95	1
73	1-hexadecylamine	3.97	0
74	heptan-2-amine	3.63	0
75	tetradecan-1-amine	4.27	0
76	N-(2-aminoethyl)ethane-1,2-diamine	3.77	0
77	1-(tricyclo[3.3.1.13,7]dec-1-yl)methanamine	2.84	0
78	4-(2-aminoethyl)benzene-1,2-diol	2.53	0
79	4-(2-aminoethyl)phenol	2.77	0
80	N-(2-aminoethyl)-N'-(3-aminopropyl)butane-1,4-diamine	2.41	0
81	N,N'-bis(2-aminoethyl)propane-1,3-diamine	2.23	0
82	3,6,9,12-tetraazatetradecane-1,14-diamine	2.54	0
83	tricyclo[3.3.1.03,7]nonan-3-amine	3.12	0
84	tricyclo[3.3.1.13,7]decan-2-amine	3.18	0
85	aminoacetic acid	2.57	0
86	methyl 2-amino-3-phenylpropanate	3.39	0
88	2-amino-3-hydroxybutanoic acid	3.21	0
89	2-amino-3-(1H-indol-3-yl)propanoic acid	3.19	0
95	2-amino-5-carbamimidamidopentanoic acid	3.15	0
96	2-aminobutanedioic acid	3.29	0
97	2,5-diamino-5-oxopentanoic acid	3.32	0

98	2-aminopentanedioic acid	3.40	0
99	2-amino-3-(1H-imidazol-4-yl)propanoic acid	3.38	0
100	2-amino-4-(methylsulfanyl)butanoic acid	3.23	0
101	2-amino-3-phenylpropanoic acid	3.29	0
102	dihydrofuran-2,5-dione	4.24	1
103	acetic anhydride	4.05	1
104	3-methylidenedihydrofuran-2,5-dione	4.04	1
105	1,4-dioxane-2,6-dione	3.99	1
106	2-benzofuran-1,3-dione	3.90	1
107	(2,5-dioxotetrahydrofuran-3-yl)acetic acid	4.03	1
	**Test set**		
2	chloro(difluoro)acetic anhydride	3.95	1
4	3,3-dimethyldihydrofuran-2,5-dione	4.11	1
12	6-bromo-1H,3H-benzo[de]isochromene-1,3-dione	3.63	0
15	5-nitro-1H,3H-benzo[de]isochromene-1,3-dione	4.11	1
24	oct-7-ene-2,3,5,6-tetracarboxylic anhydride	3.63	0
25	3a,4,7,7a-tetrahydro-2-benzofuran-1,3-dione	3.89	1
26	dibenzo[c,e]oxepine-5,7-dione	3.77	1
50	8-oxaspiro[4.5]decane-7,9-dione	4.06	0
53	3-phenyldihydro-2H-pyran-2,6(3H)-dione	4.02	0
64	3-methylbutan-1-amine	3.83	0
65	pentan-3-amine	3.81	1
69	propane-1,3-diamine	3.49	1
87	2-amino-3-hydroxypropanoic acid	3.36	1
90	2-amino-3-(4-hydroxyphenyl)propanoic acid	3.07	1
91	2-amino-3-methylbutanoic acid	3.27	0
92	2,6-diaminohexanoic acid	3.25	0
93	amino(4-chlorophenyl)acetic acid	3.06	0
94	2-aminopropanoic acid	2.90	0
108	4,7-difluoro-2-benzofuran-1,3-dione	3.91	1
109	{bis[2-(2,6-dioxomorpholin-4-yl)ethyl]amino}acetic acid	4.10	0
